# Evaluation of Oseltamivir Used to Prevent Hospitalization in Outpatients With Influenza: A Systematic Review and Meta-Analysis

**DOI:** 10.1001/jamainternmed.2023.0699

**Published:** 2023-06-12

**Authors:** Ryan Hanula, Émilie Bortolussi-Courval, Arielle Mendel, Brian J. Ward, Todd C. Lee, Emily G. McDonald

**Affiliations:** 1 Centre for Outcomes Research and Evaluation, McGill University Health Centre, Montreal, Quebec, Canada; 2 Division of Experimental Medicine, Department of Medicine, McGill University Health Centre, Montreal, Quebec, Canada; 3 Division of Infectious Diseases, Department of Medicine, McGill University Health Centre, Montreal, Quebec, Canada; 4 Division of General Internal Medicine, Department of Medicine, McGill University Health Centre, Montreal, Quebec, Canada

## Abstract

**Importance:**

Despite widespread use, summary evidence from prior meta-analyses has contradictory conclusions regarding whether oseltamivir decreases the risk of hospitalization when given to outpatients. Several large investigator-initiated randomized clinical trials have not yet been meta-analyzed.

**Objective:**

To assess the efficacy and safety of oseltamivir in preventing hospitalization among influenza-infected adult and adolescent outpatients.

**Data Sources:**

PubMed, Ovid MEDLINE, Embase, Europe PubMed Central, Web of Science, Cochrane Central, ClinicalTrials.gov, and WHO International Clinical Trials Registry were searched from inception to January 4, 2022.

**Study Selection:**

Included studies were randomized clinical trials comparing oseltamivir vs placebo or nonactive controls in outpatients with confirmed influenza infection.

**Data Extraction and Synthesis:**

In this systematic review and meta-analysis, Preferred Reporting Items for Systematic Reviews and Meta-analyses (PRISMA) reporting guidelines were followed. Two independent reviewers (R.H. and É.B.C.) extracted data and assessed risk of bias using the Cochrane Risk of Bias Tool 2.0. Each effect size was pooled using a restricted maximum likelihood random effects model. The quality of evidence was graded using the Grading of Recommendations, Assessment, Development and Evaluations (GRADE) framework.

**Main Outcomes and Measures:**

Hospitalization was pooled as risk ratio (RR) and risk difference (RD) estimates with 95% CIs.

**Results:**

Of 2352 studies identified, 15 were included. The intention-to-treat infected (ITTi) population was comprised of 6166 individuals with 54.7% prescribed oseltamivir. Across study populations, 53.9% (5610 of 10 471) were female and the mean age was 45.3 (14.5) years. Overall, oseltamivir was not associated with reduced risk of hospitalization within the ITTi population (RR, 0.79; 95% CI, 0.48 to 1.29; RD, −0.17%; 95% CI, −0.23% to 0.48%). Oseltamivir was also not associated with reduced hospitalization in older populations (mean age ≥65 years: RR, 1.01; 95% CI, 0.21 to 4.90) or in patients considered at greater risk of hospitalization (RR, 0.65; 0.33 to 1.28). Within the safety population, oseltamivir was associated with increased nausea (RR, 1.43; 95% CI, 1.13 to 1.82) and vomiting (RR, 1.83; 95% CI, 1.28 to 2.63) but not serious adverse events (RR, 0.71; 95% CI, 0.46 to1.08).

**Conclusions and Relevance:**

In this systematic review and meta-analysis among influenza-infected outpatients, oseltamivir was not associated with a reduced risk of hospitalization but was associated with increased gastrointestinal adverse events. To justify continued use for this purpose, an adequately powered trial in a suitably high-risk population is justified.

## Key Points

**Question:**

Is the administration of oseltamivir to adult and adolescent outpatients with confirmed influenza associated with a reduced risk of first hospitalization?

**Findings:**

In this systematic review and meta-analysis of 15 randomized clinical trials including 6166 patients, oseltamivir was not associated with reduced risk of first hospitalization compared with placebo or standard of care. Results were similar in a subgroup of patients considered at high risk of hospitalization; however, the CIs were wide.

**Meaning:**

An adequately powered trial in a suitably high-risk population is needed to determine who might benefit from early treatment with oseltamivir to prevent hospitalization.

## Teaser

This meta-analysis assesses the efficacy and safety of oseltamivir in preventing hospitalization among influenza-infected adult and adolescent outpatients.

## Introduction

Before the COVID-19 pandemic, influenza was one of the most clinically burdensome respiratory viruses.^1^ The US Centers for Disease Control and Prevention estimated 29 million cases, 380 000 hospitalizations, and 28 000 deaths from influenza in the US during the 2018 to 2019 season.^2^ While COVID-19 led to a temporary reduction in infections, influenza is now expected to have a resurgence.^3^ Novel strains or a rise in a relatively less immune population could trigger an influenza pandemic resembling the crises experienced in 1968 or 2009.^4^ As such, the availability of safe and effective treatments is critical to avoid overwhelming health care systems and to reduce morbidity and mortality. Indeed, a breakthrough in the COVID-19 pandemic occurred when outpatient randomized clinical trials demonstrated reductions in hospitalization and death.^5^ In contrast, despite the substantial threat that influenza poses, there are no evidence-based outpatient treatments proven to prevent the progression to hospitalization.

Oseltamivir (Tamiflu) is an antiviral that is commonly prescribed to outpatients with influenza to accelerate recovery and prevent complications. Detailing by key opinion leaders, guideline panels, and the manufacturer has even led to stockpiling of the medication as part of national pandemic responses.^6^ Yet, despite guideline recommendations,^7,8^ and millions of doses prescribed, it is unclear whether oseltamivir reduces severe complications requiring hospitalization. Three prior systematic reviews (1 independent and 2 supported by the manufacturer) have arrived at different conclusions.^9,10,11^ Since these publications, several large randomized clinical trials (RCTs) have been completed and have yet to be meta-analyzed.^12,13,14,15^ We, therefore, sought to clarify whether oseltamivir is a high-value medical treatment (achieving optimal results for patients balanced with an efficient use of resources).^16^ To do so, we conducted a systematic review and meta-analysis of RCTs of oseltamivir for the prevention of first hospitalization in adolescent and adult outpatients (efficacy) and treatment-associated adverse events (safety).

## Methods

The protocol for this systematic review and meta-analysis was prospectively registered on PROSPERO (CRD42022299030).^17^ Findings are reported following the Preferred Reporting Items for Systematic Reviews and Meta-analyses (PRISMA) reporting guideline.^18^

### Search Methods

We searched PubMed, Ovid MEDLINE, Embase, Europe PubMed Central, Web of Science, and Cochrane Central, as well as ClinicalTrials.gov and WHO International Clinical Trials Registry (eTable in Supplement 1) from inception to January 4, 2022. The central search strategy consisted of MeSH (Medical Subject Headings) terms and keywords corresponding to the subjects of “influenza,” “randomized clinical trials,” and “oseltamivir.” This was then adapted to meet each database- or registry-specific terminology. Bibliographies of included articles and relevant systematic reviews were hand searched. Unpublished Roche-sponsored clinical study reports (CSRs) were obtained from the British Medical Journal’s open database for oseltamivir.^19^ No language restrictions, filters, or limits were applied.

### Selection Criteria

Published and unpublished RCTs were included in this review. Observational studies were excluded. Each included trial compared oseltamivir at the recommended oral dosage of 75 mg twice daily for 5 days vs a nonactive control equivalent (placebo or standard of care) and reported the outcome of hospitalization. Only the first hospitalization was considered; readmissions were not counted. Study populations included outpatients aged 12 years and older diagnosed with natural influenza infections based on clinical history and laboratory evidence. Most often infection was confirmed by viral culture or polymerase chain reaction (PCR); however, in certain Roche-sponsored studies, an infection could also be established by a 4-fold rise in antibody titers at day 30.

### Study Selection and Extraction

Search results were imported to EndNote, version 9.3.3 (Clarivate), and duplicates were removed. Unique studies were uploaded to Rayyan, and 2 reviewers (R.H., É.B.C.) independently screened all titles and abstracts, removed clearly irrelevant results, selected eligible studies from full-text review, and recorded reasons for exclusion. The same reviewers then independently extracted the data from included studies using a pre-established data extraction table in Microsoft Excel, version 16.54 (Microsoft). Disagreements were resolved by consensus with a third reviewer (T.C.L.).

### Outcomes and Data Items

Extracted study characteristics included the year, number of participants, method of confirming influenza, follow-up duration, and study sponsor. Relevant participant demographics were extracted (eg, race and ethnicity, sex, influenza A or B). Missing study demographics were assumed to be unavailable.

The primary efficacy outcome was the number of first all-cause hospitalizations per treatment group in the intention-to-treat infected (ITTi) population—individuals confirmed to have influenza according to the study definition. Hospitalization was defined as the first admission to a hospital or health care center during the treatment or follow-up period, for any cause and any duration. Emergency department visits with direct discharge home were excluded. When this was not specifically reported in the ITTi population, we made up to 8 email data requests to the senior and/or corresponding author. The British Medical Journal database contains unpublished Roche CSRs.^19^ Within these, we identified hospitalized patients from the serious adverse event narratives and cross-referenced their participant identification numbers to the study’s diagnostic results to confirm their case positivity.

The primary safety outcome was the rate of any adverse event, regardless of grade, and included nausea, vomiting, diarrhea, cardiac, psychiatric, neurologic, and a composite of any gastrointestinal symptoms (eg, nausea, diarrhea, gastritis, and others). Nonindustry studies either had thresholds for reporting neurological adverse effects or did not report these at all; for example, Roche CSRs recorded neurological adverse effects (severe and nonsevere) but excluded headache and fatigue if these occurred during the 5 days of treatment and were accompanied by 1 or more additional typical symptoms of influenza (eg, myalgias). In addition, total serious adverse events (as defined by the studies) were analyzed separately whenever possible based on reporting. Adverse events were measured within the safety population (all randomized patients who received at least 1 treatment dose).

### Risk of Bias

The risk of bias for each study was assessed by 2 independent reviewers (R.H., É.B.C.) using the Cochrane Risk of Bias tool, version 2.0.^20^ Disagreements were settled by consensus, and assessments were rendered by the Risk-of-Bias Visualization tool.^21^

### Statistical Analyses

Under the Cochrane Handbook’s assumption that some heterogeneity is inevitable, a restricted maximum-likelihood random effects model was used for the meta-analyses using the meta-command in Stata, version 17.0 (StataCorp).^22^ Hospitalization was summarized as a risk ratio (RR) with 95% CIs. A continuity correction of 0.5 was used for cells with zero events. Using the *metaprop_one* module, we estimated the pooled control event rate using a generalized linear mixed model. We multiplied the pooled control event rate by (1 − risk ratio) and its 95% CIs to estimate the absolute risk difference (RD) with 95% CIs. Common adverse event types were meta-analyzed using a restricted maximum-likelihood random effects model and were reported on the RR and RD scales. If statistically significant, the number needed to harm was reported.^22^ Statistical heterogeneity was examined with the *I^2^* test whereby a value greater than 50% was considered statistically significant heterogeneity.^23^

Several secondary analyses were conducted for the outcome of hospitalization on the RR scale. First, to explore potential causes of heterogeneity, prespecified subgroup analyses were performed based on each study’s: mean population age (above and below 65 years); method of confirming influenza (PCR, viral culture, or rapid antigen); population risk level (high-risk [mean population age older than 65 years or high-risk comorbidities] vs not); and trial sponsor (Roche vs other). A subgroup analysis was also conducted based on study quality with studies grouped as either low or at greater than low risk of bias. Finally, for hospitalization, we performed a remove-one meta-analysis to ensure no singular study significantly influenced the pooled estimate and a cumulative meta-analysis to investigate for a change in efficacy over time.

Finally, since Roche studies confirmed influenza infections via viral culture as well as a 4-fold or greater increase in antibody titer and given prior studies have found oseltamivir reduces the odds of a 4-fold antibody rise by almost 20%, we conducted a post hoc analysis using the ITT populations from Roche-sponsored studies.^24^

To assess for publication bias, we visually inspected a funnel plot and performed an Egger test for asymmetry.^23^ A threshold of *P* < .10 was selected as an indicator of statistically significant publication bias.^25^

### Certainty of Evidence

Two independent reviewers (R.H., É.B.C.) evaluated the certainty of the evidence for the outcome of hospitalization using the GRADE (Grading of Recommendations, Assessment, Development and Evaluations) framework.^26^

## Results

### Search Results

**Figure 1.**
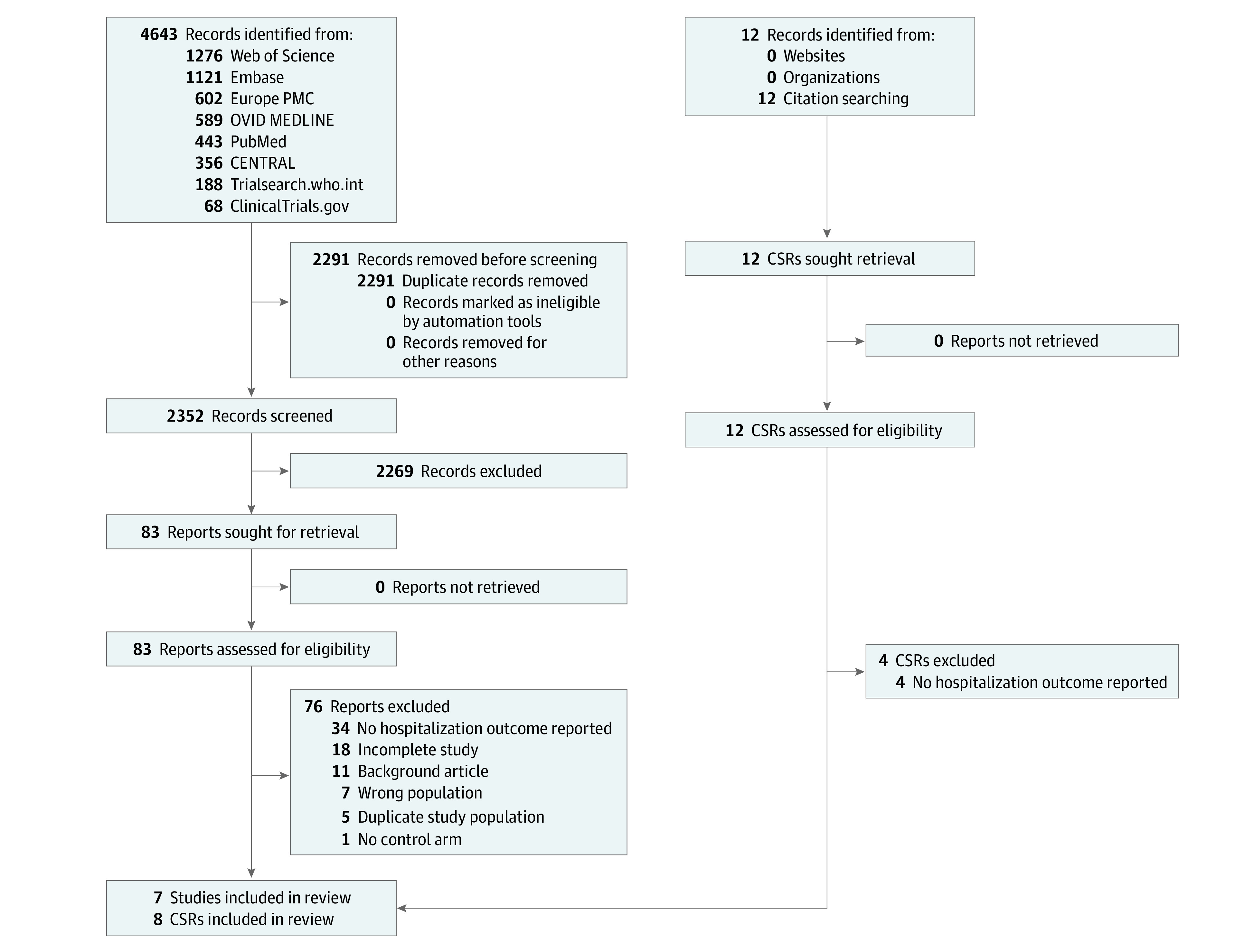
PRISMA 2020 Flow Diagram This PRISMA flow diagram was for a systematic review that included searches of databases, registers, and other sources. All records identified by other methods included CSRs provided by Roche to the British Medical Journal. Abbreviation: CSR, clinical study report.

The initial database and registry searches yielded 2352 unique studies (Figure 1). Following title and abstract screening, 2269 were excluded. From 83 full-text reviews, 76 were subsequently excluded, leaving 7 included.^12,13,14,15,27,28,29^ Hand-searching of bibliographies resulted in the inclusion of 8 additional unpublished CSRs from Roche Pharmaceuticals for a total of 15 studies in the final meta-analysis.^30,31,32,33,34,35,36,37^

### Risk of Bias

Of the 15 studies assessed, 9 (60.0%) were considered at low risk of bias, 5 (33.3%) had some concerns, and 1 (6.7%) was considered high risk (eFigure 1 in Supplement 1).

### Study and Population Characteristics

The ITTi population comprised 6166 individuals, 3324 of whom were assigned to oseltamivir (53.9%). Overall trial demographics are included in Table 1 based on the total study populations. Participants had a mean (SD) age of 45.3 (14.5) years, and 53.6% (5610 of 10 471) were female individuals. Where reported, 70.2% (4225 of 6017) of participants identified as White individuals, and 20.8% (1253 of 6017) identified as Asian individuals. A total of 60.3% (3668 of 6079) of participants were infected with influenza A. At the study level, 9 of 15 (60%) trials were sponsored by Roche and were conducted between 1998 and 2006. Across studies, the control rate of hospitalization was low (0.8%).

**Table 1.** Study Design and Population Characteristics

Source	ITTi population No. (control, oseltamivir)	Comparator	Duration of follow-up, d	Method to confirm influenza infection	Sponsor	Female, No. (%)	Male, No. (%)	Mean age	Mean weight /BMI	No. (%)	
										Race and ethnicity^a^	Influenza type A
Beigel et al,^12^ 2020	279, 277	Placebo	28	Rapid antigen	National Institute of Allergy and Infectious Diseases	347 (62.4)	209 (37.6)	NR	NR	Asian, 385 (69.2) Black, 18 (3.2) White, 150 (27.0) Other, 3 (0.5)	387 (69.6)
Hayden et al,^15^ 2018	231, 377	Placebo	21	RT-PCR	Shionogi	270 (44.4)	338 (55.6)	35.2	68.3 kg	Asian, 483 (79.4) Black, 20 (3.3) White, 100 (16.4) Other, 5 (0.8)	537 (88.3)
Ison et al,^14^ 2020	386, 389	Placebo	21	RT-PCR	Shionogi	404 (52.1)	371 (47.9)	51.5	79.3 kg	Asian, 320 (41.6) Black, 59 (7.7) White, 382 (49.6) Other, 9 (1.2)	427 (55.1)
Lin et al,^28^ 2006^b^	29, 27	Placebo	21	Viral culture	Shanghai Roche	23 (41.1)	33 (58.9)	50.3	65 kg	NR	NR
Roberts et al,^29^ 2019	7, 7	Placebo	28	Rapid antigen	GSK	3 (21.4)	11 (78.6)	34.9	30.5 (BMI)	Asian, 2 (14.3) Black, 1 (7.1) White, 11 (78.6) Other, 0 (0)	7 (50.0)
Dorkings WV15670,^31^ 1998^b^	161, 158	Placebo	21	Viral culture	Roche	239 (50.1)	238 (49.9)	37.8	74.1 kg	Asian, 21 (4.4) Black, 6 (1.3) White, 448 (93.9) Other, 2 (0.4)	302 (64.3)
Dorkings WV15671,^32^ 1999^b^	129, 124	Placebo	21	Viral culture	Roche	222 (53.1)	196 (46.9)	32.3	80.5 kg	Asian, 6 (1.4) Black, 41 (9.8) White, 341 (81.6) Other, 30 (7.2)	235 (56.1)
McGarty M76001,^30^ 2000^b^	361, 702	Placebo	21	Viral culture	Roche	808 (55.8)	639 (44.2)	35.2	78.1 kg	Asian, 25 (1.7) Black, 117 (8.1) White, 1184 (81.8) Other, 121 (8.4)	866 (59.8)
Grosse WV15707,^33^ 1999^b^	6, 6	Placebo	21	Viral culture	Roche	12 (46.2)	14 (53.8)	71.6	77.0 kg	Asian, 1 (3.8) Black, 0 (0) White, 14 (53.8) Other, 11 (42.3)	12 (46.2)
McCarvil WV15812 and WV15872,^34^ 2000^b^	133, 118	Placebo	21	Viral culture	Roche	224 (55.9)	177 (44.1)	51.8	77.8 kg	Asian, 4 (1.0) Black, 7 (1.7) White, 375 (93.5) Other, 15 (3.7)	205 (51.0)
Roche WV15819, WV15876, and WV15978,^35^ 2000^b^	254, 223	Placebo	21	Viral culture	Roche	419 (57.0)	316 (43.0)	73	74.6 kg	Asian, 2 (0.3) Black, 9 (1.2) White, 721 (98.1) Other, 3 (0.4)	449 (61.1)
Roche WV16277,^36^ 2003^b^	109, 119	Placebo	21	Viral culture	Roche	227 (50.3)	224 (49.7)	34.9	72.8 kg	Asian, 2 (0.4) Black, 3 (0.7) White, 445 (98.7) Other, 1 (0.2)	205 (45.5)
Dorkings WV15730,^37^ 1999^b^	19, 19	Placebo	21	Viral culture	Roche	28 (48.3)	30 (51.7)	35.2	72.1 kg	Asian, 2 (3.4) Black, 1 (1.7) White, 54 (93.1) Other, 1 (1.7)	36 (62.1)
Fry et al,^27^ 2014	64, 76	Placebo	Followed up until 7 d after symptoms resolved	RT-PCR	Centers for Disease Control and Prevention	563 (47.3)	627 (52.7)	NR	NR	NR	762 (65.5)
Butler et al,^13^ 2020	674, 702	Standard of care	28	PCR	European Commission’s Seventh Framework Programme	1821 (55.9)	1438 (44.1)	NR	NR	NR	948 (29.0)

Abbreviations: BMI, body mass index; ITTi, intention-to-treat infected; PCR, polymerase chain reaction; RT-PCR, reverse transcription polymerase chain reaction; NR, not reported.

^a^ In this review, “other” race and ethnicity is defined as any other race or ethnicity other than White, Black, or Asian (ie, Hispanic, American Indian, or Alaska Native) as well as those participants whose identity was not provided or collected (ie, not available).

^b^ Denotes studies that used serological testing alongside viral culture to confirm influenza diagnoses. Overall trial demographics were based on the total study populations. Demographics for the desired 12 years and older population extracted from Fry^27^ (2014) and the Butler^13^ (2020) data request were not provided, thus their entire populations are represented.

### Efficacy Outcome

**Figure 2.**
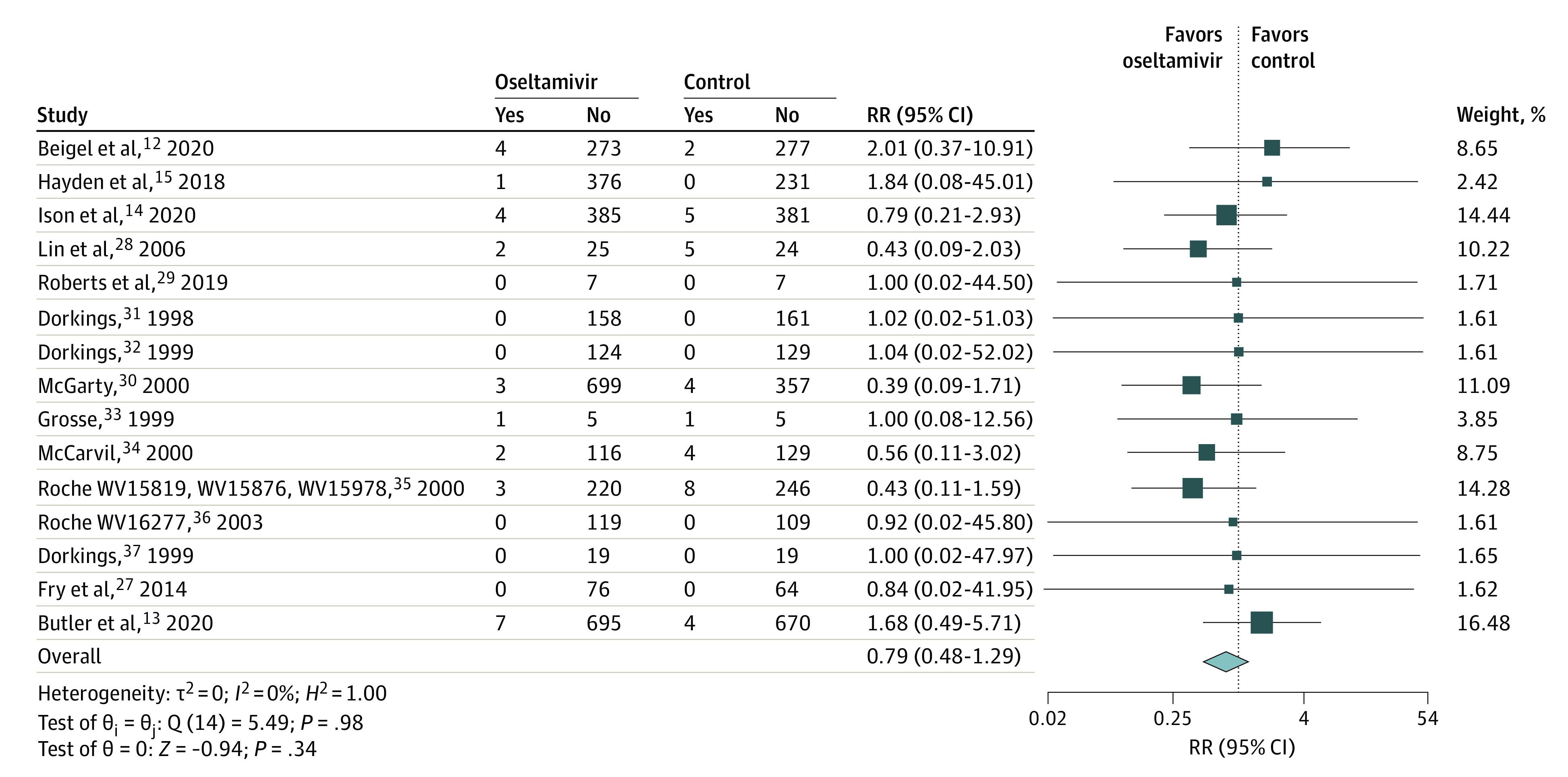
Random Effects Meta-Analysis on the Outcome of Hospitalization Within the ITTi Population Aged 12 Years and Older “Yes” indicates the number of individuals hospitalized, and “No” indicates the number of individuals who were not. Statistical heterogeneity was examined with the *I^2^* test whereby a value greater than 50% was considered statistically significant heterogeneity.

Overall, oseltamivir was not associated with reduced risk of first hospitalization in the ITTi population (RR, 0.79; 95% CI, 0.48 to 1.29; *I^2^* = 0%; RD, −0.17%; 95% CI, −0.23% to 0.48%; Figure 2).

### Subgroup Analyses

Risk of hospitalization differed substantially between the industry-sponsored and nonindustry-sponsored studies (RR, 0.50; 95% CI, 0.25-0.97 vs RR, 0.51; 95% CI, 0.26-1.01, respectively; eFigure 2A in Supplement 1). Industry-sponsored studies were also more likely to use viral culture and/or serological confirmation as opposed to modern molecular diagnostics (eFigure 2B in Supplement 1). Oseltamivir was not associated with reduced hospitalization in older populations (mean age ≥65 years: RR, 1.01; 95% CI, 0.21-4.90 vs mean age <65 years: RR, 0.73; 95% CI, 0.41-1.29; eFigure 2C in Supplement 1). Likewise, there was no observed reduction in the subgroup stratified according to patient risk (high-risk: RR, 0.85; 95% CI, 0.40-1.82 vs low-risk: 0.65; 95% CI, 0.33-1.28; eFigure 2D in Supplement 1). Subgroup analysis dichotomized by study quality (high vs low risk of bias) was also not associated with the findings (high risk of bias: RR, 0.78; 95% CI, 0.36 to 1.71 vs low risk of bias: RR, 0.78; 95% CI, 0.40-1.53; eFigure 2E in Supplement 1).

### Sensitivity Analyses

A remove-one analysis found Butler,^13^ 2020, and Roche WV15819, WV15876, and WV15978,^35^ 2000, had a greater association with the overall effect size (eFigure 3 in Supplement 1). Cumulatively, estimated efficacy decreased over time, particularly when nonindustry studies began to dominate the literature. (eFigure 4 in Supplement 1). A post hoc analysis restricted to placebo-controlled trials also found no difference in the efficacy of oseltamivir (RR, 0.68; 95% CI, 0.39-1.17).

The sensitivity analysis using the ITT populations for Roche-sponsored studies shifted the overall effect size toward the null (RR, 0.84; 95% CI, 0.55-1.29; eFigure 5 in Supplement 1). Similarly, when analyzed as a subgroup, Roche-sponsored studies also shifted closer to the null (0.68; 95% CI, 0.40-1.14). Additional subgroup analyses revealed no appreciable changes in the point estimates (eFigures 6A-6E in Supplement 1).

### Safety Outcomes

Table 2 summarizes the risk of key adverse events. Patients prescribed oseltamivir experienced significantly more nausea (RR, 1.43; 95% CI, 1.13-1.82), vomiting (RR, 1.83; 95% CI, 1.28-2.63), and a composite of gastrointestinal symptoms (RR, 1.21; 95% CI, 1.02-1.45). There was a reduced risk of diarrhea (RR, 0.76; 95% CI, 0.57-1.00). The risk of neurological disorders (RR, 1.15; 95% CI, 0.91-1.45) was not statistically higher in the oseltamivir group. Oseltamivir was not associated with an increase in serious adverse events compared with controls (RR, 0.71; 95% CI, 0.46 to 1.08).

**Table 2.** Random Effects Meta-Analyses on Adverse Events and Serious Adverse Events Within the Safety Population

Event type	Oseltamivir frequency	Placebo frequency	RR (95% CI)	Heterogeneity, %	*P* value for RR	RD (95% CI)	NNH^a^ (95% CI)
Nausea	374/3892	218/3197	1.43 (1.13 to 1.82)	39.71	.004	0.107 (0.048 to 0.167)	9.3 (6.0 to 21.0)
Vomiting	248/3120	103/2417	1.83 (1.28 to 2.63)	42.81	.001	0.164 (0.088 to 0.239)	6.1 (4.2 to 11.3)
Diarrhea	222/3841	216/3142	0.76 (0.57 to 1.00)	39.72	.05	−0.082 (−0.161 to −0.003)	−12.2 (−334.4 to −6.2)
Gastrointestinal disorders	591/2305	356/1818	1.21 (1.02 to 1.45)	39.40	.03	0.068 (0.009 to 0.126)	14.8 (7.9 to 111.7)
Cardiac disorders	29/1991	32/1505	0.69 (0.42 to 1.15)	0.00	.15	−0.107 (−0.230 to 0.017)	NA
Neurological disorders	179/2247	112/1758	1.15 (0.91 to 1.45)	0.00	.25	0.034 (−0.023 to 0.092)	NA
Psychiatric disorders	12/2247	16/1758	0.67 (0.29 to 1.53)	0.00	.34	−0.205 (−0.461 to 0.051)	NA
Serious adverse events	39/3765	49/3080	0.71 (0.46 to 1.08)	0.00	.11	−0.097 (−0.196 to 0.003)	NA

Abbreviations: NA, not applicable; NNH, number needed to harm; RD, risk difference; RR, risk ratio.

^a^ The NNH value was only reported when the primary effect was statistically significant.

### Publication Bias

Visual inspection of the funnel plot revealed asymmetry; however, the Egger test was not statistically significant (*P* = .68; eFigure 7 in Supplement 1).

### GRADE Certainty of Evidence

It was concluded with moderate-certainty evidence that oseltamivir had little to no effect on hospitalization. Although all included studies were RCTs directly evaluating oseltamivir, there was imprecision in the estimates due to wide variability between study results, not all studies were placebo-controlled, and some studies were at risk of bias. Although the present analysis stratified by risk of bias produced similar estimates, these aforementioned factors decreased the present study’s certainty from strong to moderate.

## Discussion

This systematic review and meta-analysis of oseltamivir for the outpatient treatment of laboratory-confirmed influenza included approximately 3400 more patients than prior analyses.^9,11^ Despite this, oseltamivir was not associated with significantly reduced hospitalization in general or in prespecified high-risk subgroups. Interestingly, the subgroup analysis limited to industry-sponsored studies did suggest a reduced risk of hospitalization in the ITTi population. One possible explanation is that the industry studies used viral culture, and it is possible that the PCR used in modern trials detects milder cases with lower viral loads and/or residual nonviable virus when compared with viral culture. Another possibility includes Roche’s allowance of a 4-fold rise in antibody responses to confirm infection. There is evidence that oseltamivir may reduce seroconversion and therefore patients hospitalized with negative serology in the oseltamivir group may have been misclassified as noninfected.^24^ A final explanation could be the lower prevalence of oseltamivir resistance when industry studies were conducted. Since that time, a greater than 10-fold rise in resistance has been observed (0.32% in the early 2000s to 3.56% between 2008 and 2013).^38,39^ Nonetheless, many of these industry trials only came to light after a legal challenge, and it is reasonable to look at the evidence in total. It was reassuring that there was no increase in severe adverse events observed despite oseltamivir being strongly associated with an increased risk of gastrointestinal adverse effects (nausea and vomiting).

Based on the present analyses, it appeared unlikely that administration of oseltamivir to a general outpatient population had a meaningful effect on serious influenza-related outcomes culminating in hospitalization. That said, it should be noted that the rate of hospitalization was exceedingly low, with a control event rate of 0.81% (95% CI, 0.29%-2.20%). For oseltamivir to continue to be part of a viable influenza response with respect to preventing severe complications, future studies should focus on identifying the groups of higher-risk participants, with laboratory-confirmed influenza, who may derive benefit. Conducting an adequately powered trial would require a large sample size; however, given millions have received oseltamivir, such a trial does not seem unreasonable. As examples, we modeled 2 possible scenarios. First, if the risk of hospitalization is very low (eg, approximately a 1% rate as observed in the general population),^40^ a study of 30 716 participants would be required to demonstrate a 30% relative risk reduction with 80% power and 2-sided α = .05. By comparison, to conduct a trial focused on patients at greater risk of hospitalization, (eg, the 2% event rate among this population in the present analysis), 15 232 participants would be required. To succeed at recruitment, such trials would need to either take place during an epidemic or pandemic year or over several years of seasonal influenza. Although the required sample size is large, it is potentially achievable; PANORAMIC (Platform Adaptive Trial of Novel Antivirals for Early Treatment of COVID-19 in the Community) recruited 25 783 participants for early outpatient COVID-19 treatments between December 8, 2021, and April 27, 2022.^41^

### Limitations

The present meta-analysis had several limitations. First, we analyzed CSRs together with published and nonindustry trials; these differed in the time frame over which they took place, the mechanism for diagnosing infection, and the granularity of the data included. Second, the mean age of the patients was young (mid-40s) and the rate of hospitalization was low. This might have limited the power to detect an effect but also implies that any missed effect would have a very high number needed to treat. We also chose a priori to analyze first hospitalization, whereas others have included readmissions.^24^ This difference, and the inclusion of the newer trials, should be factored in when comparing these results with prior analyses. Similarly, we excluded patients assigned to high-dose oseltamivir as this is not the approved dosing and therefore is less clinically relevant. Third, although the present search methods were robust, there is always the possibility that some studies, particularly unpublished ones, were missed. Fourth, we did not study symptomatic improvement, which, along with the associated outcome of return to work, could be important during a pandemic. Prior studies have reported small improvements in symptom duration (16.8-25.2 hours).^9,11^ Whether this decrease is meaningful when compared with medication costs, an increase in nonsevere adverse events, and the opportunity cost of missing out on the discovery of more effective therapies is a topic of study for health care economists and could be discussed on an individual patient basis. Finally, we excluded observational data from the present analysis. While observational data can contribute larger numbers of patients, and the data can be more affordable and faster to access, it is also subject to substantial biases (eg, immortal time bias, confounding by indication, residual confounding), which make it unsuitable for evaluating medication efficacy even with the most robust statistical methods.^42^

To our knowledge, this is the first systematic review and meta-analysis focusing on oseltamivir specifically for the reduction of all-cause hospitalization, an important outcome to prevent overwhelming health care systems. Given many of the adverse effects of oseltamivir can overlap with symptoms of influenza, we thought it important to study all-cause hospitalization, rather than influenza-related hospitalizations, which would overlook complications related to the medication’s adverse effects. This is particularly relevant for older high-risk adults where seemingly mild gastrointestinal adverse effects might still increase the risk of hospitalization through anorexia and dehydration.

## Conclusions

Based on the available RCT data in this systematic review and meta-analysis, there is a lack of convincing evidence that oseltamivir reduces serious complications in outpatients with influenza, although its use is associated with an increase in nonsevere gastrointestinal adverse events. This meta-analysis provides important data for clinicians, patients, and policy makers to contextualize the evidence and inform guidelines. Future research should focus on the conduct of an adequately powered placebo-controlled trial in a suitably high-risk population.

## Notes

Supplement 1. **eTable 1.** Search strategies **eFigure 1.** Visualization of the risk of bias **eFigure 2.** Random effects subgroup analyses on the outcome of hospitalization **eFigure 3.** Random effects remove-one sensitivity analysis on the outcome of hospitalization **eFigure 4.** Random effects cumulative meta-analysis on the outcome of hospitalization **eFigure 5.** Random effects sensitivity analysis on the outcome of hospitalization **eFigure 6.** Random effects sensitivity subgroup analyses on the outcome of hospitalization **eFigure 7.** Publication bias funnel plot on the outcome of hospitalization

Supplement 2.
Data Sharing Statement
