# Supplementary material for: Evaluation of Oseltamivir Used to Prevent Hospitalization in Outpatients With Influenza: A Systematic Review and Meta-Analysis
Source: JAMA Intern Med. 2023 Jun 12;184(1):18–27. doi: 10.1001/jamainternmed.2023.0699 (PMC10262060; doi:10.1001/jamainternmed.2023.0699)
Supplement: Supplement 1. — eTable 1. Search strategies eFigure 1. Visualization of the risk of bias eFigure 2. Random effects subgroup analyses on the outcome of hospitalization eFigure 3. Random effects remove-one sensitivity analysis on the outcome of hospitalization eFigure 4. Random effects cumulative meta-analysis on the outcome of hospitalization eFigure 5. Random effects sensitivity analysis on the outcome of hospitalization eFigure 6. Random effects sensitivity subgroup analyses on the outcome of hospitalization eFigure 7. Publication bias funnel plot on the outcome of hospitalization [file jamainternmed-e230699-s001.pdf]

Supplemental Online Content

Hanula R, Bortolussi-Courval E, Mendel A, Ward BJ, Lee TC, McDonald EG. Evaluation of oseltamivir used to prevent hospitalization in outpatients with influenza: a systematic review and meta-analysis. *JAMA Intern Med.* Published online June 12, 2023. doi:10.1001/jamainternmed.2023.0699

eTable 1. Search strategies

eFigure 1. Visualization of the risk of bias

eFigure 2. Random effects subgroup analyses on the outcome of hospitalization

eFigure 3. Random effects remove-one sensitivity analysis on the outcome of hospitalization

eFigure 4. Random effects cumulative meta-analysis on the outcome of hospitalization

eFigure 5. Random effects sensitivity analysis on the outcome of hospitalization

eTable 6. Random effects sensitivity subgroup analyses on the outcome of hospitalization

eFigure 7. Publication bias funnel plot on the outcome of hospitalization

This supplemental material has been provided by the authors to give readers additional information about their work.

© 2023 Hanula R et al. *JAMA Internal Medicine.*

Supplemental eTable 1. Search strategies

Ovid Medline

#	Search	Results
1	Influenza A virus/	22175
2	Influenza B virus/	4528
3	Influenza*.mp.	145690
4	flu.mp.	15249
5	Influenza, Human/	54694
6	1 or 2 or 3 or 4 or 5	153306
7	randomized controlled trial.pt.	566020
8	controlled clinical trial.pt.	94836
9	random*.ab.	1271612
10	placebo.ab.	227506
11	trial.ab.	596535
12	groups.ab.	2340038
13	7 or 8 or 9 or 10 or 11 or 12	3624063
14	Exp animals/ not humans.sh.	4997861
15	13 not 14	3114255
16	Oseltamivir/	3151
17	Oseltamivir*.mp.	4908
18	Tamiflu*.mp.	518
19	16 or 17 or 18	4986
20	6 and 15 and 19	589

Embase

#	Search	Results
1	Influenza virus A/ or Influenza A virus/	24615
2	Influenza virus B/ or Influenza B virus/	7198
3	influenza B/ or influenza/ or influenza B/	84196
4	influenza*.mp.	223242

© 2023 Hanula R et al. *JAMA Internal Medicine.*

5	flu*.mp.	3178533
6	1 or 2 or 3 or 4 or 5	3362888
7	Randomized controlled trial/	708162
8	Controlled clinical trial/	465854
9	random*.ti,ab.	1792299
10	randomization/	93918
11	Intermethod comparison/	282433
12	placebo.ti,ab.	344616
13	(compare or compared or comparison).ti.	593655
14	((evaluated or evaluate or evaluating or assessed or assess) and (compare or compared or comparing or comparison)).ab.	2491458
15	(open adj label).ti,ab.	96390
16	((double or single or doubly or singly) adj (blind or blinded or blindly)).ti,ab.	261129
17	double blind procedure/	196834
18	parallel group*1.ti,ab.	29275
19	(crossover or cross over).ti,ab.	117233
20	((assign$ or match or matched or allocation) adj5 (alternate or group$1 or intervention$1 or subject$1 or participant$1)).ti,ab.	380014
21	(assigned or allocated).ti,ab.	447816
22	(controlled adj7 (study or design or trial)).ti,ab.	409163
23	(volunteer or volunteers).ti,ab.	273118
24	human experiment/	572817
25	trial.ti.	364746
26	or/7-25	5815599
27	(random$ adj sampl$ adj7 (cross section$ or questionnaire$1 or survey$ or database$1)).ti,ab. Not (comparative study/ or controlled study/ or randomi?ed controlled.ti,ab. Or randomly assigned.ti,ab.)	9055
28	Cross-sectional study/ not (randomized controlled trial/ or controlled clinical study/ or controlled study/ or	307281

© 2023 Hanula R et al. *JAMA Internal Medicine.*

	randomi?ed controlled.ti,ab. or control group$1.ti,ab.)	
29	(((case adj control$) and random$) not randomi?ed controlled).ti,ab.	19729
30	(Systematic review not (trial or study)).ti.	207099
31	(nonrandom$ not random$).ti,ab.	17850
32	Random field$.ti,ab.	2691
33	(random cluster adj# sampl$).ti,ab.	1430
34	(review.ab. and review.pt.) not trial.ti.	987121
35	we searched.ab. And (review.ti. or review.pt.)	41495
36	update review.ab.	122
37	(databases adj4 searched).ab.	50138
38	(rat or rats or mouse or mice or swine or porcupine or murine or sheep or lambs or pigs or piglets or rabbit or rabbits or cat or cats or dog or dogs or cattle or bovine or monkey or monkeys or trout ot marmoset$1).ti. and animal experiment/	1146778
39	Animal experiment/ not (human experiment/ or human/)	2410619
40	or/27-29	3948480
41	26 not 40	5160620
42	oseltamivir/	13392
43	Oseltamivir*.mp.	14030
44	tamiflu*.mp.	2317
45	42 or 43 or 44	14079
46	6 and 41 and 45	1121

Cochrane CENTRAL

#	**Search**	**Results**
1	(Influenza A virus):ti,ab,kw	3139
2	(Influenza B virus):ti,ab,kw	1282
3	(Influenza*):ti,ab,kw	9988
4	(flu*):ti,ab,kw	111122

© 2023 Hanula R et al. *JAMA Internal Medicine.*

5	MeSH descriptor: [Influenza, Human] this term only	2902
6	(Influenza A*):ti,ab,kw	8029
7	(Influenza B*):ti,ab,kw	7399
8	1 or 2 or 3 or 4 or 5 or 6 or 7	119243
9	(controlled clinical trial):pt	331021
10	(randomized controlled trial):pt	545182
11	(random*):ab	950488
12	(placebo):ab	308565
13	(trial):ab	504148
14	(groups):ab	517708
15	9 or 10 or 11 or 12 or 3 or 14	1265774
16	MeSH descriptor: [Oseltamivir] this term only	252
17	(oseltamivir*):ti,ab,kw	577
18	(tamiflu*):ti,ab,kw	86
19	16 or 17 or 18	584
20	8 and 15 and 19	356

PubMed

#	Search	Results
1	(((((influenza a virus) OR (influenza b virus)) OR (influenza*)) OR (flu*)) OR (influenza a*)) OR (influenza b*)	153288
2	(((((randomized controlled trial[Publication Type]) OR (controlled clinical trial[Publication Type])) OR (placebo[Title/Abstract])) OR (clinical trials as topic [MeSH Terms])) or (random*[Title/Abstract])) OR (trial[Title])	1804352
3	(animals[MeSH Terms]) NOT (humans[MeSH Terms])	4937458
4	2 not 3	1659482
5	((oseltamivir[MeSH Terms]) OR (oseltamivir*)) OR (tamiflu*)	4942
6	1 and 4 and 5	443

© 2023 Hanula R et al. *JAMA Internal Medicine.*

Europe PMC

#	Search	Results
1	((((TS=(influenza*)) OR TS=(flu*)) OR TS=(influenza a virus)) OR TS=(influenza b virus))	4110241
2	(((((TS=(randomized controlled trial)) OR TS=(controlled clinical trial)) OR AB=(random*)) OR AB=(placebo)) OR AB=(trial)) OR AB=(groups)	7021918
3	((TS=(oseltamivir)) OR TS=(tamiflu*)) or TS=(oseltamivir*)	5870
4	1 and 2 and 3	1276

Web of Science

#	Search	Results
1	TOPIC: (influenza a virus) OR TOPIC: (influenza b virus) OR TOPIC: (influenza*) OR TOPIC: (flu*)	4115204
2	TS=(controlled clinical trial) OR TS=(randomized controlled trial) OR AB=(random*) OR AB=(placebo) OR AB=(groups) OR AB=(trial)	7035023
3	TOPIC: (Oseltamivir*) OR TOPIC: (tamiflu*)	5881
4	1 and 2 and 3	1276

Clinicaltrials.gov

#	Search	Results
1	Condition or disease: Influenza OR Influenza A OR Influenza B, Other terms: Oseltamivir OR Tamiflu Study Type: Interventional (Clinical Trial)	68

World Health Organization International Clinical Trials Registry Platform (trialsearch.who.int)

#	Search	Results
1	influenza AND (oseltamivir OR tamiflu)	188

© 2023 Hanula R et al. *JAMA Internal Medicine.*

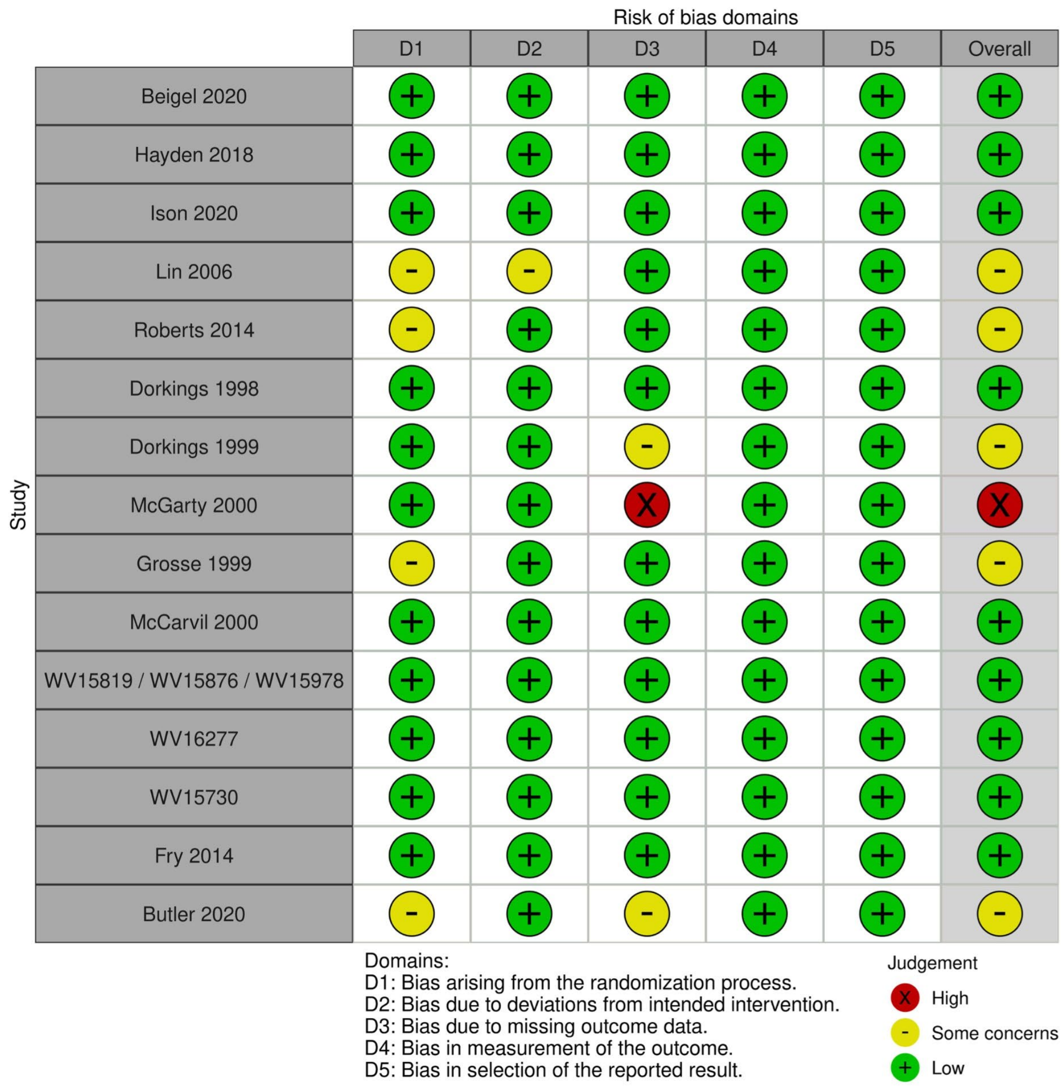

Supplemental eFigure 1. Visualization of the risk of bias. Determined using the Cochrane Risk of Bias Tool 2.0.

© 2023 Hanula R et al. *JAMA Internal Medicine.*

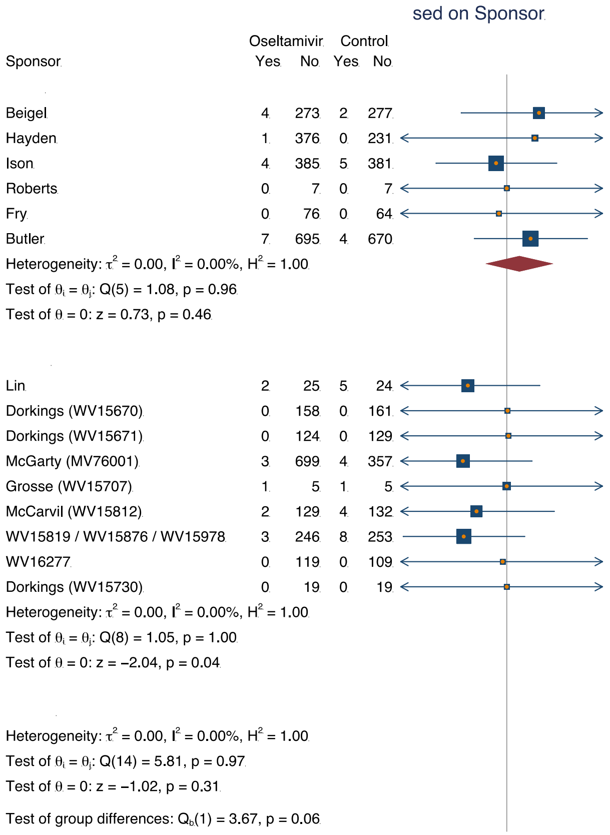

41 **A)**

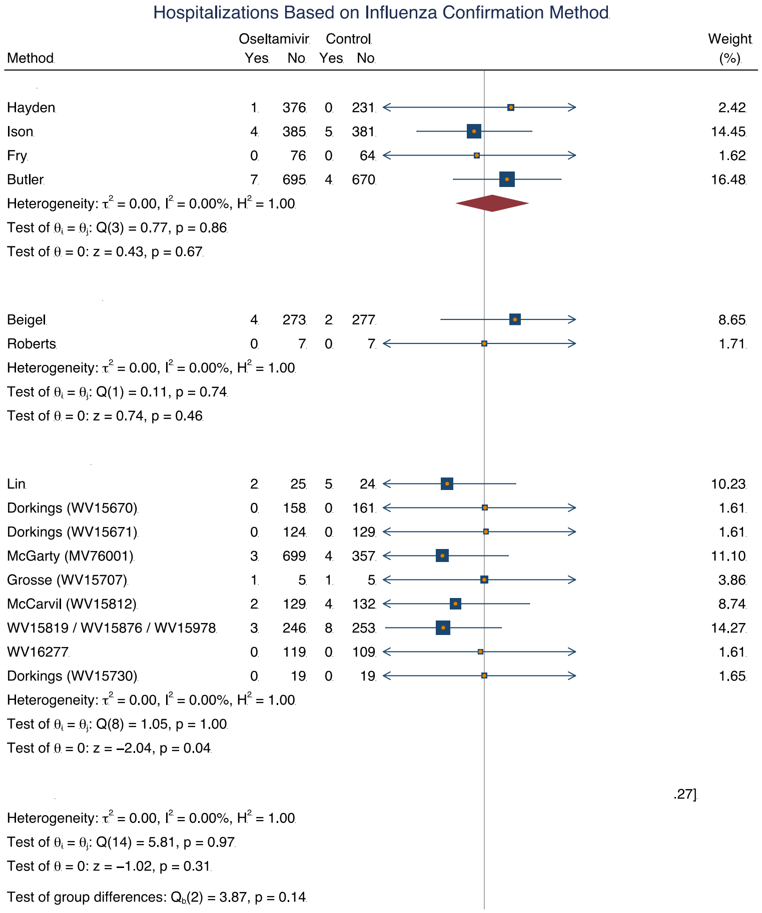

B)

© 2023 Hanula R et al. *JAMA Internal Medicine.*

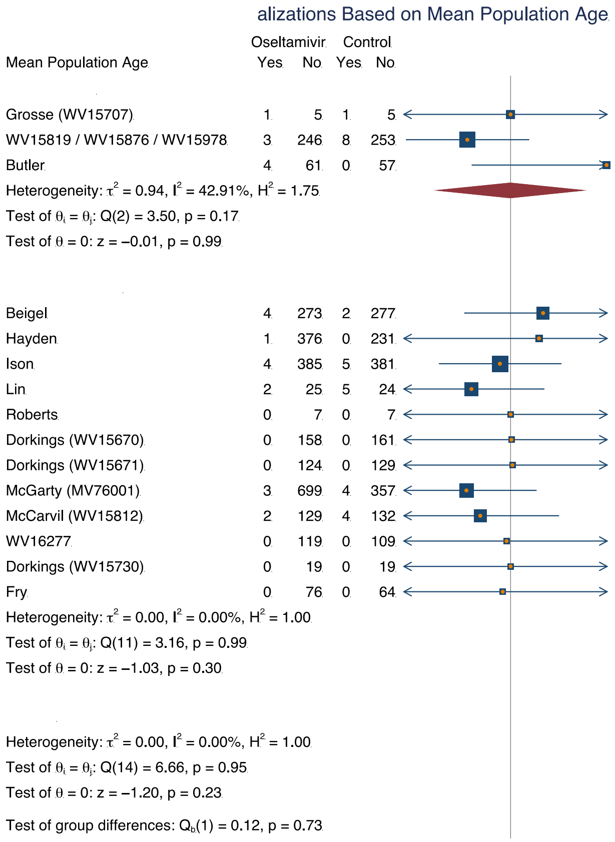

42 **C)**

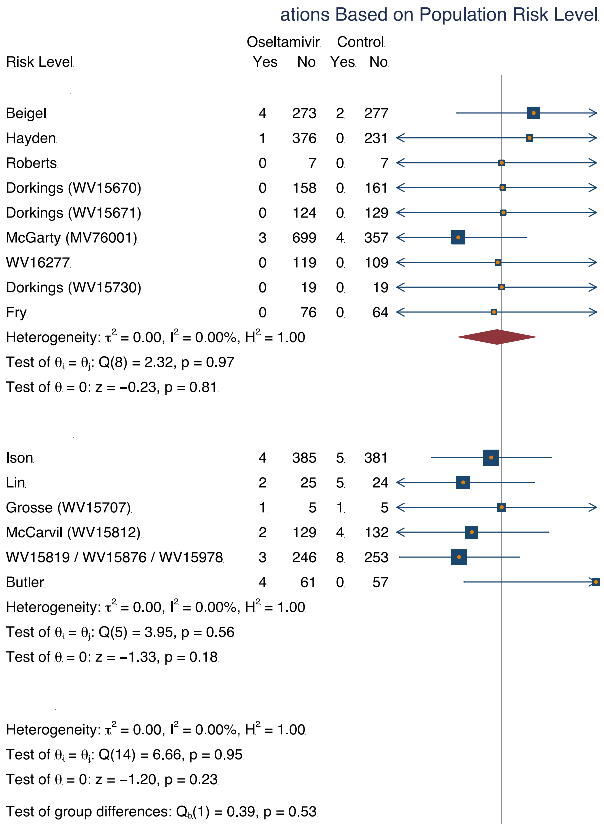

D)

© 2023 Hanula R et al. *JAMA Internal Medicine.*

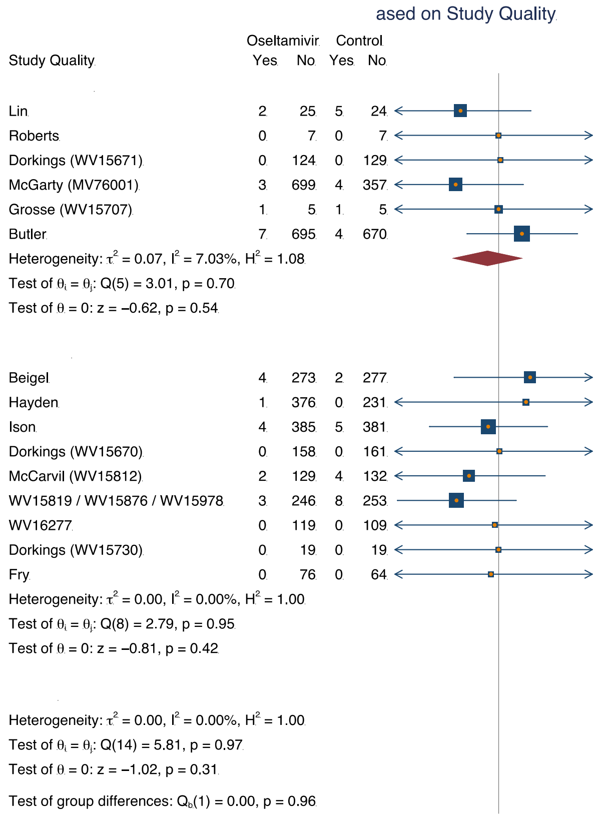

E)

Supplemental eFigure 2. Random effects subgroup analyses on the outcome of hospitalization. Studies were stratified according to A) Study Sponsor, B) Method of Confirming Influenza Infections, C) Mean Study Population Age, D) Population Risk Level, and E) Study Quality. Yes = number of individuals hospitalized, No = number of individuals who were not. Note: Butler's (2020) 12+ ITTi data provided via data request was used for the subgroup estimates in forest plots A, B, and E. However, the supplemental 65+ ITTi data also provided was used for the subgroup estimates in forest plots C and D.

© 2023 Hanula R et al. *JAMA Internal Medicine.*

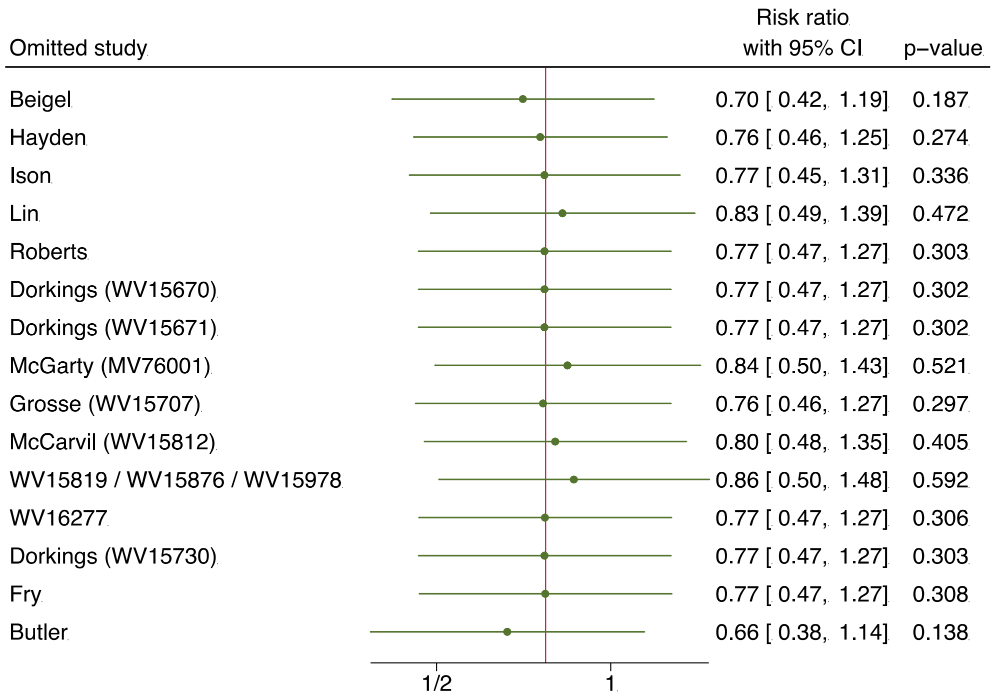

Supplemental eFigure 3. Random effects remove-one sensitivity analysis on the outcome of hospitalization.

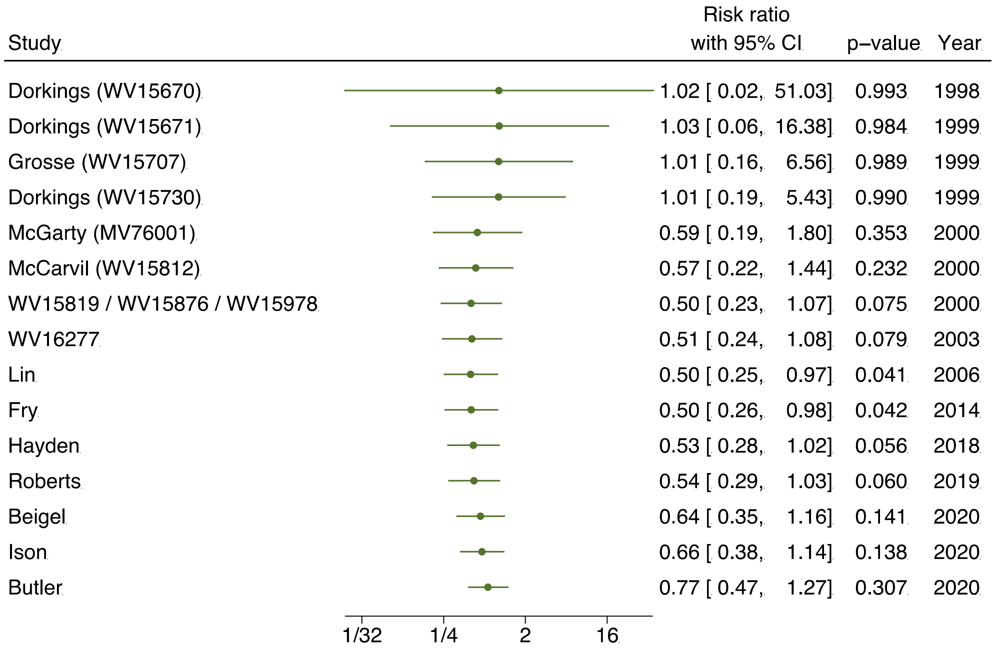

Supplemental eFigure 4. Random effects cumulative meta-analysis on the outcome of hospitalization.

© 2023 Hanula R et al. *JAMA Internal Medicine.*

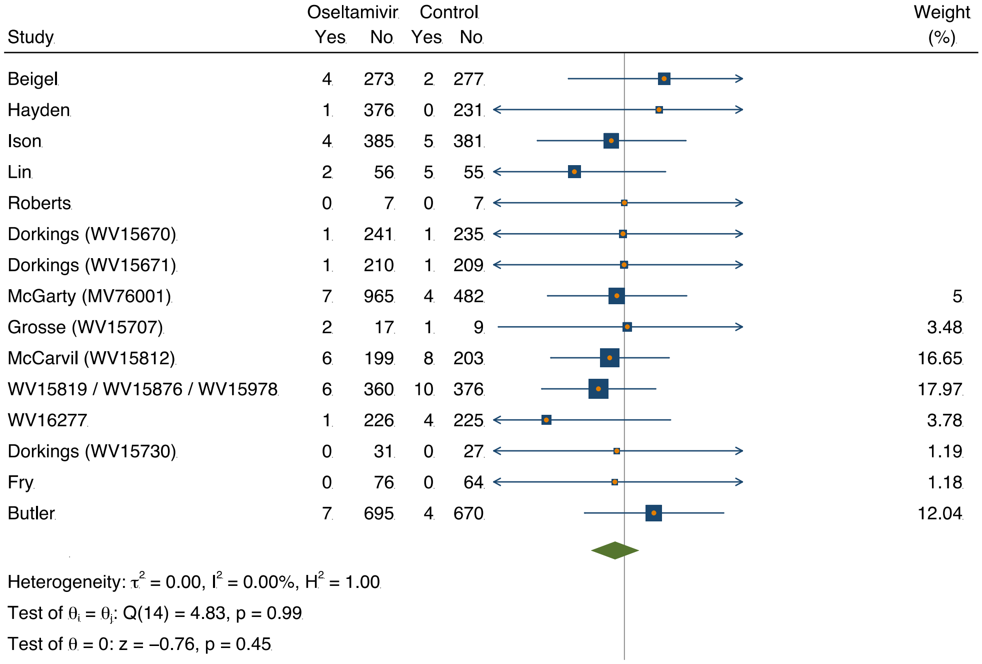

Supplemental eFigure 5. Random effects sensitivity analysis on the outcome of hospitalization. Outcome data was based on the 12+ ITT populations for Roche-sponsored studies and 12+ ITTi populations for non-industry studies. Yes = number of individuals hospitalized, No = number of individuals who were not.

© 2023 Hanula R et al. *JAMA Internal Medicine.*

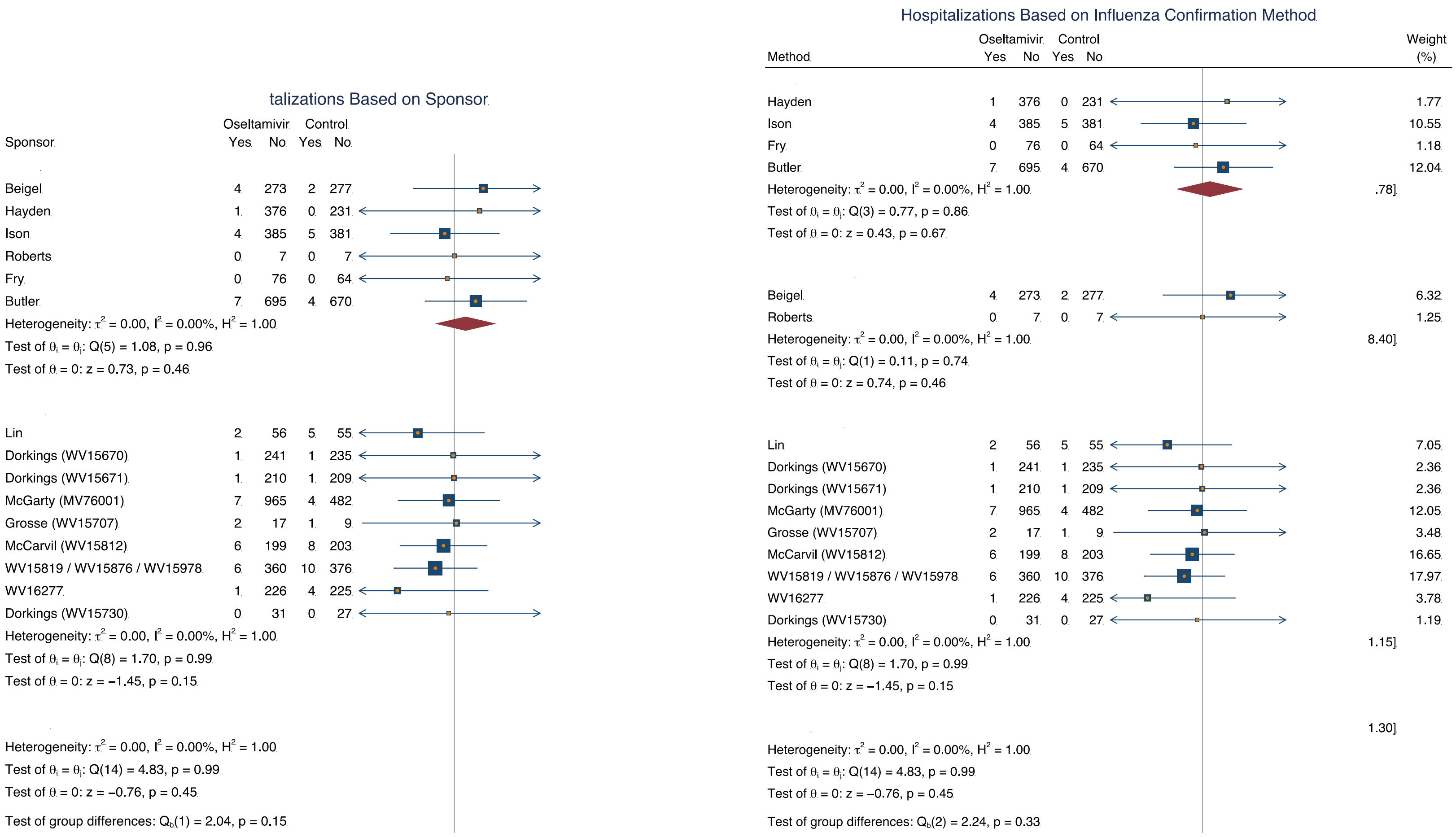

63 **A)** **B)**

© 2023 Hanula R et al. *JAMA Internal Medicine.*

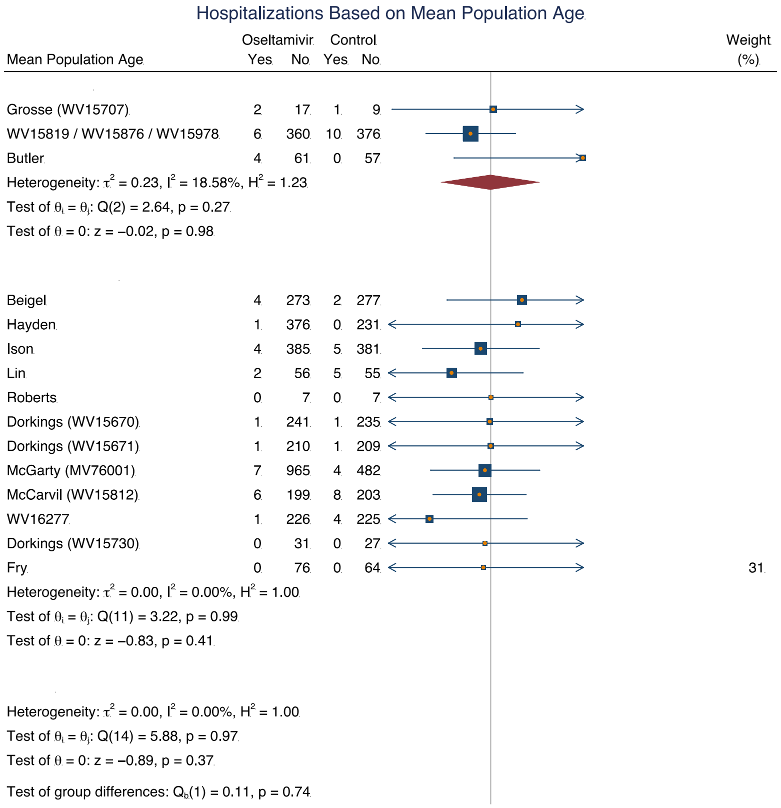

64 **C)**

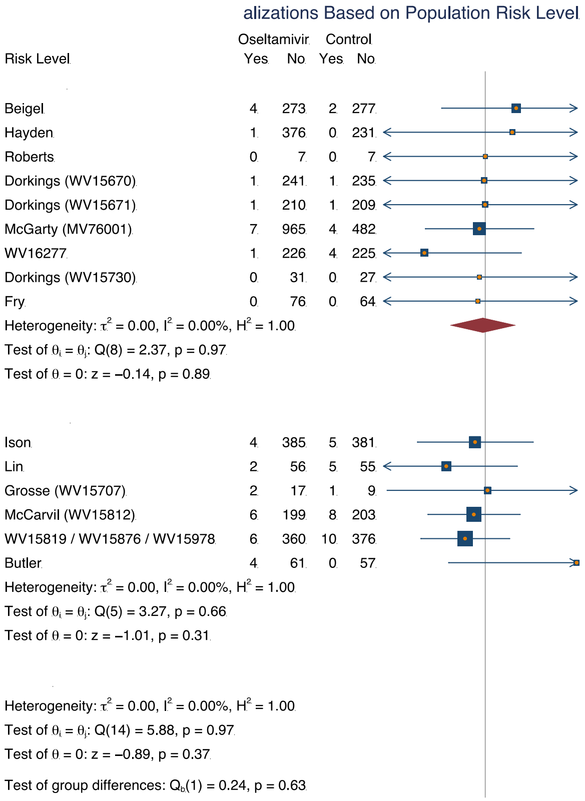

D)

© 2023 Hanula R et al. *JAMA Internal Medicine.*

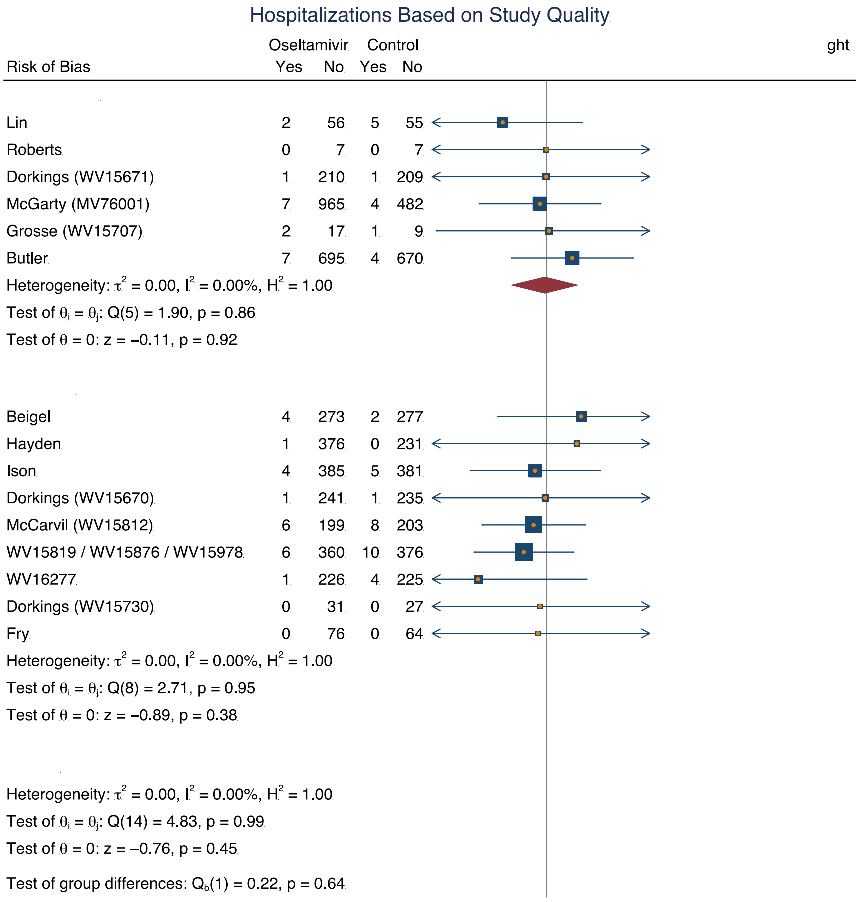

E)

Supplemental eFigure 6. Random effects sensitivity subgroup analyses on the outcome of hospitalization. Outcome data was based on the ITT populations for Roche-sponsored studies and ITTi populations for non-industry studies. Studies were stratified according to A) Study Sponsor, B) Method of Confirming Influenza Infections, C) Mean Study Population Age, D) Population Risk Level, and E) Study Quality. Yes = number of individuals hospitalized, No = number of individuals who were not. Note: Butler's (2020) data was utilized the same as in eFigure 2.

© 2023 Hanula R et al. *JAMA Internal Medicine.*

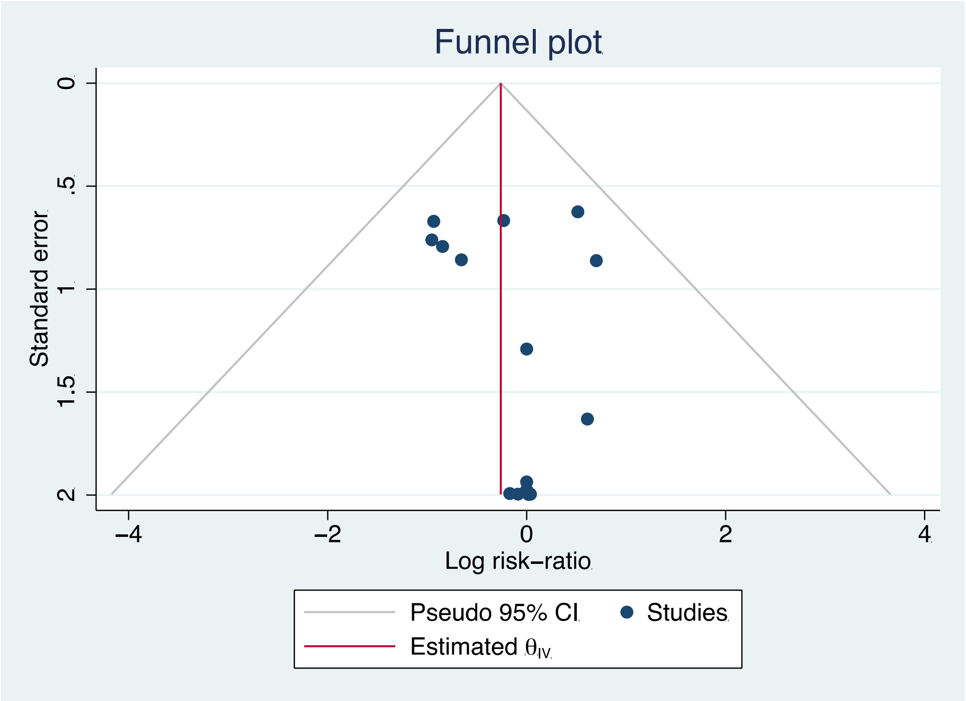

Supplemental eFigure 7. Publication bias funnel plot on the outcome of hospitalization.

© 2023 Hanula R et al. *JAMA Internal Medicine.*
