# Supplementary material for: Evaluation of Oseltamivir Used to Prevent Hospitalization in Outpatients With Influenza: A Systematic Review and Meta-Analysis
Source: JAMA Intern Med. 2023 Jun 12;184(1):18–27. doi: 10.1001/jamainternmed.2023.0699 (PMC10262060; doi:10.1001/jamainternmed.2023.0699)
Supplement: Supplement 2. — Data Sharing Statement [file jamainternmed-e230699-s002.pdf]

Data Sharing Statement

Hanula. Evaluation of Oseltamivir Used to Prevent Hospitalization in Outpatients With Influenza. *JAMA Intern Med*. Published June 12, 2023. doi:10.1001/jamainternmed.2023.0699

Data
Data available: Yes
Data types: Other (please specify)
Additional Information: Collected outcome and demographic data from each of the included studies within our meta-analysis
How to access data: ryan.hanula@mail.mcgill.ca
When available: With publication

Supporting Documents
Document types: None

Additional Information
Who can access the data: Anyone requesting the data
Types of analyses: For any purpose
Mechanisms of data availability: With investigator support
